# Sleep problems among sexual minorities: a longitudinal study on the influence of the family of origin and chosen family

**DOI:** 10.1186/s12889-021-12308-0

**Published:** 2021-12-21

**Authors:** Antony Chum, Andrew Nielsen, Celine Teo

**Affiliations:** 1 Dalla Lana School of Public Health, University of Toronto, Toronto, Canada; 2 Department of Applied Health Sciences, Brock University, St. Catharines, Canada; 3 MAP Centre for Urban Health Solutions, Unity Health Toronto, Toronto, Canada

**Keywords:** Lesbian, Gay, Bisexual, Sexual minorities, Sleep, Family support, Chosen family

## Abstract

**Background:**

There is growing evidence that lesbian, gay, and bisexual (LGB) adults experience more sleep problems than the general population. As LGB individuals experience a significantly greater risk of family rejection and low family support, our study investigates the role of family support as a potential determinant of LGB sleep problems over a prolonged period, and whether friend support (i.e. chosen family) can mitigate the effect of low family support. Given the importance of sleep on mental and physical health, study results may help shed light on persistent health disparities across sexual orientations.

**Methods:**

Our sample included 1703 LGB individuals from the *UK Household Longitudinal Study (UKHLS)*. Mixed-effect logistic regressions were used to estimate the effect of family and friend support on the development of sleep problems after 24 months while controlling for potential confounders. A modified Pittsburgh Sleep Quality Index was used to measure 1) presence of any sleep problems, 2) short sleep duration, and 3) poor sleep quality.

**Results:**

Family support at baseline was independently associated with all sleep problems in our study after 24-months: 1 SD increase in family support was associated with a 0.94 times lower risk of sleep problems (95% C.I = 0.90-0.98), a 0.88 times lower risk of short sleep duration (95% C.I = 0.81-0.95), and a 0.92 times lower risk of sleep quality (95% C.I = 0.93-0.98). Support from one’s chosen family (proxied by friend support) did not mitigate the effects of low family support on sleep problems.

**Conclusions:**

Our study found a consistent effect of family support across all sleep outcomes along with evidence of a persistent effect after 24 months. Our findings point to the importance of targeting family support in designing interventions aimed at reducing LGB sleep problems.

**Supplementary Information:**

The online version contains supplementary material available at 10.1186/s12889-021-12308-0.

## Background

### Family support as a social determinant of sleep

Families play an integral role in supporting the health and wellbeing of individuals, and several studies have shown that a lack of family support in the general population has been linked to poor sleep outcomes [1–3]. A US-based study involving 175 adults found that the presence of unsupportive parents was associated with a decrease in sleep quality as measured by the Pittsburgh Sleep Quality Index [2]. In another US study (*n* = 2871), having a supportive family relationship reduced the likelihood of monthly sleep problems by 18% and weekly sleep problems by 24% [4]. Moreover, in a 2018 meta-analysis of social support and sleep [1], the authors noted the lack of longitudinal evidence to understand the long-term impacts of family support on sleep. The importance of family support on sleep may be even greater for LGB individuals compared to the general population. A longitudinal study found that family support was the only source of social support (among family, friend, and partner support) uniquely associated with LGB mental health [5]. However, while cross-sectional association between family support and sleep has been established in the general population in the studies cited above, we are unclear of the strength of its influence on the LGB population and whether it has long-lasting impacts.

Prior research has provided evidence that being socially excluded from one’s family is linked to negative health outcomes for LGB individuals due to the increased allostatic load, emotion dysregulation, and cognitive processes that confer risk of poor mental health outcomes [6–9]. LGB-related discrimination and social exclusion can increase proximal stressors in the form of poor self-image, internalized homophobia, fear of rejection, and concealing one’s sexual orientation according to Hatzenbuehler’s psychological mediation framework [10, 11]. Previous studies among youth also provide evidence that family support has a critical role in shaping early LGB identity development [12, 13] with sustained effects on mental wellbeing throughout one’s adulthood [5]. These studies highlight the importance of family as a locus of LGB minority stress, which in turn can impact LGB sleep outcomes [14]. In Meyer’s landmark paper on LGB minority stress [6], the author makes a similar argument that family exclusion, which he argues to be an important source of minority stress, has led to traumatizing effects on LGB individuals, leading to experiences of nightmares, sleep disturbances, diarrhea, and restlessness. Given that sleep problems are associated with cardiovascular disease [15], anxiety and depression [16], type-2 diabetes [17], and all-cause mortality [17], investigating the effects of family support on LGB sleep problems may help us better understand the determinants of persistent mental and physical health disparities across sexual orientations [18, 19].

### Lack of family support among LGB individuals

Compared to heterosexuals, LGB individuals are more likely to be rejected by their families and experience a lack of family support [20, 21]. In a US-based study (*n =* 224), two-thirds of sexual minority individuals reported experiencing family rejection because of their orientation [13]. LGB youth from the Longitudinal Study of Australian Children reported lower levels of family support compared to heterosexuals (0.43 standard deviations (SD) lower than the mean support of heterosexuals) [22]. In a study of Western Europe including 754 LGBT individuals, 51% reported discrimination and/or a lack of support from their families [23]. In a recent UK survey of 5375 LGBT individuals, only 46% reported that they were able to be open about their sexual orientation to their family [24]. A lack of family support for LGB individuals has been linked to a range of detrimental health outcomes, including higher risk of mood disorders [13, 21], lower levels of psychological well-being [25], increased risks of suicide-related behaviours [13, 26, 27], a higher likelihood of experiencing internalized homophobia [28], and depression [13, 21]. On the other hand, the contribution of family rejection/lack of family support to sleep problems among LGB individuals is not well understood.

### Disparities in sleep problems across sexual orientations

Disparities in sleep problems between the LGB and general population have been documented in prior studies [25, 29–31]. LGB individuals are more likely to report short sleep duration and other sleep problems, including snoring, sleep latency, and low sleep quality, compared to the general population [17, 32, 33]. A population-based study examining 169,392 adults found that a greater number of sexual minorities reported less than 5 h of sleep compared to heterosexual individuals (17.3% vs 12.2%) [10]. In a US population-based study, gay men had 32% higher (adjusted) prevalence of trouble falling asleep and a 22% higher prevalence of waking up feeling unrested compared to heterosexual men, whereas bisexual women had a 43% higher prevalence of trouble falling asleep compared to heterosexual women [29].

A recent literature review noted that while sleep disparities for LGB individuals are well documented, there is a dearth of research investigating possible social determinants of sleep disparities across sexual orientations [34]. The authors found a possible relationship between stress and sleep problems for LGB individuals, where one study explored a pathway identifying family rejection as a possible determinant of LGB sleep problems [35]. The study found that sexual minority men have increased sleep difficulties which were mediated by poor relationships with their fathers: sexual minority men had 0.09 SD lower paternal relationship quality compared to heterosexual men (*p* < 0.001), where each SD decrease in paternal relationship quality was associated with 0.04 SD (*p* < 0.05) increase in sleep difficulties. However, the authors noted the limitations of their cross-sectional study and the need for longitudinal studies to justify causal inferences.

The purpose of this study is to build on the prior literature by: 1) investigating prolonged impact of family support on LGB sleep problems, and 2) understanding the role of family support in the larger context of LGB-specific social support systems. More specifically, many LGB individuals who come out to their families of origin (e.g., biological or legal family) may encounter some form of rejection or negative reaction, which can lead to a greater reliance on extrafamilial relationships for support (e.g., advice, guidance, resources) [36–39]. These extensive friendship networks, also known as one’s *chosen family*, have been found to buffer the negative effects of homophobia and rejection [40] by substituting support from one’s family of origin. While family rejection can have an immediate effect on LGB sleep problems (captured in the prior cross-sectional study [25]), the association is unknown in the medium term (e.g. after 2+ years), as LGB people may adjust to family rejection by relying more on their chosen family for support. A number of qualitative studies show that sexual minorities consider chosen family members to be the most prominent sources of support in their lives [41–43]. However, a recent study of 175 LGBTQ+ individuals found that support from one's family of origin was associated with psychological wellbeing, and the effects of the chosen family were fully attenuated in adjusted models [39]. Research on the chosen family as a social determinant of LGB health is scant, and none have been linked to LGB sleep problems. Further research is needed to understand the interplay of support from family of origin and chosen family in the development of LGB sleep problems over the life-course.

Our study uniquely contributes to the literature by 1) further clarifying the role of family support as a determinant of LGB sleep problems, and 2) investigating whether there is evidence that the chosen family can mitigate the effects of low family support on sleep among LGB individuals. Unlike prior studies that have largely focused on the short-term or cross-sectional associations of family support and sleep, we take advantage of a longitudinal design to investigate its prolonged impact (after 24 months). We ask the following research questions: 1) does family support have a prolonged effect on sleep problems for sexual minorities after adjusting for individual- and environmental-level confounders? 2) If so, can the friendship networks of LGB individuals (i.e., their chosen family) help mitigate the negative effects of family rejection on sleep?

## Methods

The United Kingdom Household Longitudinal Survey (UKHLS) is a nationally representative longitudinal household panel study which began in 2009. The study included a random stratified sample of approximately 40,000 households in the first wave and has been surveying individuals aged 16 or older every 1-2 years. Further information on recruitment, locations, relevant dates, and follow-up can be found on the UKHLS website. Our sample included 1703 sexual minority individuals of the UKHLS who participated in the sleep questionnaires of UKHLS. Sexual orientation was captured by asking participants to identify which of the following best describes their sexual orientation: “heterosexual or straight”, “gay or lesbian”, “bisexual”, “other”, or “prefer not to say”. Previous LGB studies have used this measure of sexual orientation as a valid instrument against multi-item instruments with high agreement (kappa statistic of 0.89) [44]. Participants with other sexual orientations (e.g. asexual, pansexual) may have self-identified as “other”. Furthermore, the survey did not include a question on whether participants were transgender. A key advantage of longitudinal study is the opportunity to link prospectively measured exposure variables to subsequent individual-level health outcomes; therefore, the key exposure and control variables are captured at waves 2 and 5, and they are used to estimate sleep outcomes at waves 4 and 7 respectively. The use of time-lagged dependent variables is a common strategy to investigate long-term and lasting impacts of key exposures on a health outcome in longitudinal research [45]. The original survey was approved by the Ethics Committee of the University of Essex. The exemption for ethical approval was granted by the Brock University Research Ethics Board.

### Exposure measures

To measure social support, we drew on the UKHLS family and friend support instruments, which have been previously validated and found to have high internal consistency (α = 0.84) and predictive validity for general mental health and psychiatric distress as measured by General Health Questionnaire-12 [5]. Since the chosen family has been defined as one’s extensive friendship network, rather than relationships established through blood and legal ties [36], we used the friend support questionnaire to measure it. The family and friend support instruments were administered in waves 2 and 5, and were constructed from six questions (i.e. understands the way you feel, can rely on them, can talk about worries, criticizes you, lets you down, gets on your nerves). Each question was rated from 1 (not at all) to 4 (a lot). Scores were summed for each participant at each time point and standardized.

### Outcome measure

Sleep problems were measured in waves 4 and 7, which allowed us to examine the impact of family support 24 months after exposure. This study has 3 outcomes (sleep problems) that were based on the Pittsburgh Sleep Quality Index (PSQI), all of which were self-reported based on participant experience in the past month: the presence of sleep dysfunction, short sleep duration, and poor sleep quality. The PSQI has shown to have high validity and reliability with adult respondents (α = 0.86) [16, 46]. Based on a previous study [30] the participant was considered to have a sleep dysfunction if they indicated ‘yes’ to any of the following more than three times a week: a) unable to sleep within 30 min (sleep latency), b) cough or snore loudly, c) waking up mid-sleep or too early, d) trouble staying awake during the day. Short sleep duration was defined as sleeping less than 6 hours on average in the past month [31], and is associated with increased risk of obesity, cardiovascular disease, stroke, and depression [47]. Sleep quality was rated on a 4-point scale from very bad to very good and responses were dichotomized into good or bad quality sleep.

### Control variables

We controlled for social factors that have been shown to influence LGB sleep problems at the neighbourhood-level based on prior literature, which included perceptions of neighbourhood safety [30]. To account for differences at the neighbourhood level, we used Buckner’s Neighbourhood Cohesion index, which includes questions on neighbourhood safety, sense of belonging, trust in neighbours, and attraction to neighbourhood. The questions were rated on a scale of 1 (strongly agree) to 5 (strongly disagree). These responses were totaled and derived to reflect a final cohesion score of 1 for lowest cohesion to 5 for highest cohesion. Cronbach’s alpha for waves 2 and 5 were 0.86 and 0.88, which demonstrates a high level of internal consistency. Prior studies have shown that the index is predictive of mental health in LGB individuals [11] and minority populations [48].

Regional differences have also been found to impact LGB sleep problems: a prior study found that state-level differences in support for same-sex marriage were associated with poor sleep among lesbian women [49]. Therefore, we controlled for regional-level fixed effects (i.e., London, North East, North West, East Midlands, West Midlands, East of England, South East, South West, Yorkshire and the Humber, Wales, Scotland, and Northern Ireland) to adjust for difference in level of LGB acceptance and availability of resources. Furthermore, we controlled for LGB density at the regional-level, which was derived using the Annual Population Survey [50].

The following individual-level characteristics were included as potential confounders: net individual income in the last month (£), highest level of education attained (no education, GSCE/A-Level, degree/higher degree, or other), ethnicity (British white, other European white, and non-white), physical health (measured using Short Form-12 physical component score, PCS-12), adverse health condition (presence of a diagnosed mental or physical chronic condition in the last 12 months), current smoker and/or heavy drinker (defined as 26 units for men, 18 for women, in a single sitting at least once in the past month) [51], frequent use of sleep aid medication (more than once a week), year fixed-effect, sexual orientation, natural log of age, and gender (man/woman). Since the UKHLS only included a single item question for sex (with the only available choices being male or female), sex was used as a proxy for gender in our study.

### Statistical analysis

Mixed-effect logistic regressions were used to estimate the longitudinal associations between the main exposures (family and friend support) and a time-lagged dependent variable (sleep outcomes) using a maximum likelihood algorithm while controlling for all potential confounders described above. Three fully adjusted models are presented in this paper, with model 1 for sleep dysfunction, model 2 for short sleep duration, and model 3 for poor sleep quality (see Supplementary Tables 1 and 2 for unadjusted associations between family support/friend support and sleep outcomes). For all models, serial autocorrelation is dealt with through individual-level random intercepts. Survey weights were included to minimize bias from selection of participants and non-responses. We tested for interaction between our 2 main exposure variables, family and friend support, in all models. Models were estimated using the PGLM (panel generalized linear models) package in RStudio version 1.2.5001. For all results, we followed a standardized method of converting odds ratios to relative risk [52]. To deal with missing data, we analyzed the full, incomplete dataset using maximum likelihood estimation (MLE). This method does not impute any data, instead it uses each case’s available data to compute MLE based on the distributional properties of the statistical model. The likelihood is computed separately for those cases with missing data and those with complete data on all variables. These two likelihoods are maximized together to find the estimates. Prior studies provided evidence that MLE performed similarly to multiple imputation in its ability to provide unbiased parameter estimates and standard errors in empirical and simulation studies with missing data [53, 54].

## Results

In our study sample (*n* = 1703), 43.1% had sleep dysfunction, 16.4% had short sleep duration, and 20.0% had poor sleep quality. The average lag time between the measure of the main exposures (family and friend support) and sleep outcomes is 24 months (SD = 1.5 months). Family and friend support had high internal consistency, with Cronbach alpha of these measures at α = 0.88 and α = 0.89 respectively. Baseline characteristics of the study sample are displayed in Table 1. In unadjusted analyses, both family and friend support reduced the risk of experiencing sleep dysfunction, short sleep duration, and poor sleep quality (at *p* < 0.05) (Supplementary Tables 1 and 2).

**Table 1:** Baseline characteristics of study cohort (*n* = 1703)

	Overall ***N*** = 1703 (%)	Has sleep dysfunction ***n*** = 734	Short sleep duration ***n*** = 280	Poor sleep quality ***n*** = 342
**Family support**				
Mean (SD)	− 0.266 (1.08)	−0.367 (1.14)	− 0.469 (1.21)	−0.439 (1.17)
Missing	247 (14.5%)	104 (14.2%)	39 (13.9%)	54 (15.8%)
*p*-value		< 0.001	0.001	0.001
**Friend Support**				
Mean (SD)	−0.069 (0.977)	− 0.145 (1.05)	− 0.214 (1.05)	− 0.165 (1.02)
Missing	241 (14.2%)	103 (14.0%)	39 (13.9%)	54 (15.8%)
*p*-value		0.02	0.003	0.035
**Gender**				
Man	796 (46.7%)	322 (43.9%)	122 (43.6%)	132 (38.6%)
Woman	907 (53.3%)	412 (56.1%)	158 (56.4%)	210 (61.4%)
Missing	0 (0.0%)	0 (0.0%)	0 (0.0%)	0 (0.0%)
*p*-value		0.02	0.208	< 0.001
**Sexual Orientation**				
Homosexual	589 (34.6%)	234 (31.9%)	79 (28.2%)	107 (31.3%)
Bisexual	611 (35.9%)	263 (35.8%)	101 (36.1%)	135 (39.5%)
Other	503 (29.%)	237 (32.3%)	100 (35.7%)	100 (29.2%)
Missing	0 (0.0%)	0 (0.0%)	0 (0.0%)	0 (0.0%)
*p*-value		0.001	0.019	0.001
**Qualifications**				
No secondary	189 (11.1%)	109 (14.9%)	47 (16.8%)	44 (12.9%)
Secondary	791 (46.4%)	323 (44.0%)	130 (46.4%)	164 (48.0%)
Post secondary	526 (30.9%)	202 (27.5%)	66 (23.6%)	88 (25.7%)
Others	153 (9.0%)	84 (11.4%)	35 (12.5%)	38 (11.1%)
Missing	44 (2.6%)	16 (2.2%)	2 (0.1%)	8 (2.3%)
*p*-value		0.147	0.091	0.357
**Substance use**				
Yes	1179 (69.2%)	531 (72.3%)	195 (69.6%)	247 (72.2%)
No	524 (30.8%)	203 (27.7%)	85 (30.4%)	95 (27.3%)
Missing	0 (0.0%)	0 (0.0%)	0 (0.0%)	0 (0.0%)
*p*-value		0.459	0.445	0.715
**Marital Status**				
Single	968 (56.8%)	387 (52.7%)	133 (47.5%)	194 (56.7%)
Married/Civil Partner	500 (29.4%)	230 (31.3%)	90 (32.1%)	87 (25.4%)
Separated/Divorced/Widowed	179 (10.5%)	98 (13.4%)	50 (17.9%(	54 (15.8%)
Missing	56 (3.3%)	19 (2.6%)	7 (2.5%)	7 (2.0%)
*p*-value		0.003	< 0.001	0.211
**Ethnicity**				
British white	1332 (78.2%)	610 (83.1%)	214 (76.4%)	290 (84.8%)
European white	48 (2.8%)	16 (2.2%)	7 (2.5%)	3 (0.0%)
Non-white	323 (19.0%)	108 (14.7%)	59 (21.1%)	49 (14.3%)
Missing	0 (0.0%)	0 (0.0%)	0 (0.0%)	0 (0.0%)
*p*-value		< 0.001	0.193	0.009
**Had a chronic mental or physical condition for the past 12-months**				
Yes	600 (32.4%)	322 (43.9%)	141 (50.4%)	161 (47.1%)
No	1250 (67.5%)	411 (56.0%)	139 (49.6%)	181 (52.9%)
Missing	2 (0.0%)	1 (0.0%)	0 (0.0%)	0 (0.0%)
*p*-value		< 0.001	< 0.001	< 0.001
**Diagnosed with clinical depression**				
Yes	296 (17.4%)	181 (24.6%)	80 (28.6%)	108 (31.6%)
No	1407 (82.6%)	553 (75.4%)	200 (71.4%)	234 (68.4%)
Missing	0 (0.0%)	0 (0.0%)	0 (0.0%)	0 (0.0%)
*p*-value		< 0.001	< 0.001	< 0.001
**SF-12 PCS score**				
Mean (SD)	51.01 (9.821)	49.3 (11.2)	47.7 (12.0)	48.7 (12.2)
Missing	0 (0.0%)	0 (0.0%)	0 (0.0%)	0 (0.0%)
*p*-value		< 0.001	< 0.001	< 0.001
**Neighbourhood cohesion**				
Mean (SD)	3.397 (0.816)	3.380 (0.836)	3.273 (0.876)	3.301 (0.887)
Missing	0 (0.0%)	0 (0.0%)	0 (0.0%)	0 (0.0%)
*p*-value		0.244	0.003	0.009

All *p*-values are from unadjusted logistic regression between sleep problem and sample characteristics

We found evidence that family support was independently associated with all three of the tested sleep problems after 24-months in LGB individuals. In fully-adjusted regression models (see Table 2), each standard deviation increase in family support was associated was associated with a 0.94 times lower risk of sleep problems (95% C.I = 0.90-0.98), a 0.88 times lower risk of short sleep duration(95% C.I = 0.81-0.95), and a 0.92 times lower risk of sleep quality (95% C.I = 0.93-0.98), with results from models 1, 2, and 3, respectively. Friend support was not independently associated with any sleep problems in fully adjusted models. In separate models, we tested for effect modification of friend support by family support, but we did not find evidence that the effect of friend support on sleep was different for those at different levels of family support.

**Table 2:** Fully adjusted results of mixed-effect logistic regressions predicting the effect of family support on sleep problems

	Model 1: Sleep Dysfunction (≧1 sleep problem)	Model 2: Sleep Duration (< 6 h of sleep)	Model 3: Sleep Quality (poor sleep quality)
	Risk Ratio (95% CI)		
**Family support (z-score)**	0.94 (0.90 - 0.98)**	0.88 (0.81 - 0.95)**	0.92 (0.87 - 0.98) *
**Friend support (z-score)**	0.98 (0.01 - 1.03)	0.95 (0.88 - 1.04)	0.99 (0.92 - 1.06)
**Gender & sexuality**			
Homosexual man	*Reference group*		
Homosexual woman	0.94 (0.79 - 1.09)	0.75 (0.52 - 1.07)	1.01 (0.78 - 1.30)
Bisexual man	0.95 (0.81 - 1.11)	1.09 (0.77 - 1.48)	1.00 (0.75 - 1.28)
Bisexual woman	1.15 (1.00 - 1.29)*	1.11 (0.83 - 1.47)	1.22 (0.97 - 1.50)
Other man	1.03 (0.86 - 1.21)	1.13 (0.78 - 1.57)	0.99 (0.73 - 1.30)
Other woman	1.15 (1.00 - 1.31)	1.52 (1.13 - 1.98) **	1.30 (1.01 - 1.62)*
**Neighbourhood cohesion**	0.94 (0.89 - 1.00)	0.82 (0.72 - 0.93)**	0.88 (0.88 - 0.97)*

All models above included additional controls for region-level fixed-effect, year fixed-effect, wave fixed-effect, household fixed-effect, ethnicity, marital status, age (in natural log units), personal income (in £1000), highest qualification, SF-12 PCS, chronic health problems, clinical depression, frequent use of sleep aid, and substance use

*p < 0.05, **p < 0.011

In addition, bisexual women were independently associated with a 1.15 times greater risk of experiencing sleep dysfunction (95% C.I = 1.00-1.29). Other women (sexual minority but not homosexual or bisexual) were also independently associated with 1.52 times greater risk of experiencing short sleep duration (95% C.I = 1.13-1.98) and a 1.30 times greater risk of poor sleep quality (95% C.I = 1.01-1.62). While neighbourhood cohesion was not the main focus of this study, we also found that a one-point increase in neighbourhood cohesion (on a 5-point scale) was associated with 0.82 times (95% CI = 0.72-0.93) lower risk of short sleep duration, and 0.88 times (95% CI = 0.81-97) lower risk of poor sleep quality.

## Discussion

### The importance of family support on sleep problems

Our study provides evidence that family support (i.e., support from one’s family of origin) has a prolonged effect on the sleep problems of sexual minorities, where higher levels of family support were associated with reductions in the risk of sleep dysfunction, short sleep duration, and poor sleep quality. In contrast, support from the chosen family did not have prolonged effects on the sleep problems with sexual minorities, and we did not find evidence that the chosen family was able to mitigate the negative effects of family rejection on sleep problems.

While the effects of family support in our study may seem modest in size (e.g., 12% reduction in risk of short sleep duration with 1 SD increase in support), the effects are consistent across the 3 sleep outcomes we tested. Moreover, the salience of low family support among sexual minorities means that even modest effect sizes add up to a significant LGB health issue: approximately half of LGB individuals (based on Western European studies) experience low family support and rejection [28, 29], and up to two-third of LGB individuals in the US reported family rejection based on their sexual orientation [13]. The lack of family support in LGB individuals has been examined for its impact on a range of health outcomes [13, 21, 26, 55], but this is the first study to investigate its effects on sleep problems, and more importantly, establish the persistent nature of the problem. While prior cross-sectional studies have investigated the association between low family support and sleep problems [1–3], and our results are consistent a prior cross-sectional study that found that LGB sleep difficulties are mediated by parental relationships [35], the lack of longitudinal studies (in the general or LGB population) has prevented researchers and clinicians from understanding the prognosis of sleep problems as a result of low family support. By establishing that the association between sleep problems and low family support persists over 24 months, we provide evidence that LGB sleep problems resulting from low family support are likely not going to “go away on their own” through psychological adjustment or adaptation.

Historically, advocacy organizations including PFLAG [56] (Parents, Family and Friends of Lesbians and Gays) in the US (1973), and FFLAG [57] (Families and Friends of Lesbians and Gays) in the UK (1956) were established to address the abuse (e.g. verbal, physical, property crimes) and rejection experienced by sexual minorities perpetrated by their family of origin. Despite advancements in LGB human rights such as marriage equality and anti-discrimination laws [58], there are still persistent health disparities across sexual orientations, many of which may be the result of family rejection [13]. Our study results (covering the period of 2012-2017) highlights the harms (with regards to sleep problems) as a result of widespread unsupportive families among sexual minorities that continues today, and the ongoing need to improve the capacity of these organizations to improve LGB family support through sustained funding from local and national governments. In addition, our findings emphasize the need for evidence-based family oriented interventions for sexual minorities such as the *Family Acceptance Project* [55], which help families accept and support their LGBT children. The *Family Acceptance Project* has developed a screening tool that helps identify and measure family and caregiver behaviours that are predictive of negative health and mental health outcomes for LGBT youths.

### The influence of the chosen family on sleep problems

Our study is also the first to provide evidence that support from one’s chosen family (i.e. friend support) did not reduce sleep problems among sexual minorities. Furthermore, there is no evidence that support from one’s chosen family can replace family support (i.e. the effect of friend support on sleep was not significant at any level of family support). Our results are consistent with a prior longitudinal study association between friend support and general mental health that was fully attenuated after adjusting for family support [5], and a qualitative study that chosen family is more likely to complement rather than replace the support of biological families [43]. Therefore, support from one’s chosen family alone may not be enough to completely overcome the sleep problems associated with having an unsupportive family of origin. However, these findings are nonetheless notable, given the myriad qualitative studies that emphasize the integral role that chosen family can play in an LGB individual’s life, whether the chosen family is additive or completely substitutes the support of their family of origin [41–43]. Additionally, when the chosen family is complementary to their family of origin, sexual minorities tend to place more value in approval received from their friends over that of their family, and feel generally more supported by friends [42].

### The protective effect of neighbourhood cohesion

Our findings also suggest that neighbourhood cohesion has a protective effect against sleep problems in sexual minorities, which is consistent with prior studies that identified the same protective effect among Native [59] and Latino [60] populations in the US. While there is currently no evidence of the effect of neighbourhood cohesion on sleep among sexual minorities, a prior study has found that neighbourhood cohesion has a positive influence on the mental health of LGB individuals [11]. As neighbourhood cohesion is the social connections shared with one’s neighbours, the sense of belonging that develops through these relationships may be of added value for sexual minorities as LGB individuals tend to face social rejection from other areas of their lives [23, 43]. Future studies should investigate how interventions to increase neighbourhood cohesion (e.g. promoting safe spaces for LGB individuals and LGB-friendly community events) may reduce sleep problems among sexual minorities, in order to understand how to design neighbourhoods that promote sexual minority health.

### Strengths and limitations

Although it is well-documented that sexual minorities have worse sleep outcomes compared to the general population, there are few studies examining possible social determinants of LGB sleep problems [35, 61]. While a previous study suggested the possible influence of family support on LGB sleep problems [35], a causal relationship could not be established from its cross-sectional design (i.e. it is possible that sleep problems may have exacerbated family support issues). Our study uses a repeated measures analysis with time-lagged dependent variables (24-months lag) to establish temporal ordering of the family support exposure and sleep outcomes, which provides stronger evidence that family support should be considered a social determinant of sleep for LGB individuals. While a number of studies have demonstrated that a lack of family support is linked to a range of health outcomes in LGB individuals (i.e. higher rates of mood disorders, suicide-related behaviours [27], risky sexual behaviours, and illicit substance usage [13], our study provides further evidence that it can also contribute to sleep problems.

There are a few study limitations that would affect the generalizability of our results: 1) A single item question was used to capture the sexual orientation each participant identified with, but did not include questions on sexual attraction and behaviours. This may lead to respondents selecting a sexual orientation that does not truly represent the complex nature of their sexuality, which can then lead to misclassification. The availability of the “other” category may have helped reduce misclassification: since it is available to participants who did not feel they belonged to the categories of heterosexual, homosexual, or bisexual. 2) The UKHLS survey does not include any information on the participant’s disclosure of their sexual orientation to their family members, which could be considered a potential confounder. 3) The UKHLS survey does not ask about gender identity (i.e. does not include transgender and non-binary as valid choices), which limits the generalizability of our results and they may not be applicable to transgender individuals. 4) Sleep problems were based on participant self-reports which may affect the reliability of this measure; however, the PSQI is a well validated instrument used in prior studies to identify sleep problems. 5) There was approximately 14% missing data for family and friend support which may have led to selection bias, however our statistical models used MLE to account for the missing data which has been shown to be comparable to using multiple imputation [53]. 6) Another source of selection bias could be that self-identification of LGB on the UKHLS survey may be influenced by potential family rejection, resulting in some individuals misidentifying instead as heterosexual or preferring not to say. It is important to note that sexual orientation concealment (e.g. not disclosing one’s sexual orientation for the fear of social and family rejection) has been linked to reduced physical and mental health in prior studies [62, 63], and increases the risk of experiencing sleep disturbances [6]. Moreover, these individuals with concealed sexual identities have been found to have lower levels of family support [64]. Therefore, by excluding this group, our findings may have been biased towards the null. 7) While there were no evidence from our study that the effect of family support on sleep problems differed by sexual orientation (e.g. homosexual vs. bisexual), we cannot rule out this possibility given the small cell sizes in some categories, which could have resulted in a Type-II error. Future studies that are sufficiently powered to investigate the effect modification by gender and sexual orientation groups are required to rule out the possibility of effect heterogeneity of family support across these categories.

### Implications for practice

Findings from our study support the development of routine protocols for screening of sleep problems in LGB patients in primary care settings. For clinicians working with LGB people who experience sleep problems, they can investigate the patient’s level of family support as a potential cause of these issues, which can then inform further clinical or social interventions. For example, psycho-social supports which aim to improve sexuality support within the family unit such as through family counselling or culturally-informed psychotherapy, can target the root issue of rejection and lack of support that drives the sleep disparity among sexual minorities.

## Supplementary Information

**Additional file 1.**

## Data Availability

Waves 1-9 of UKHLS data are available via registration with the UK Data Service (beta.ukdataservice.ac.uk/datacatalogue/series/series?id = 2000053). This dataset is open to the public.

## Abbreviations

LGB: Lesbian, gay, bisexual
UKHLS: United Kingdom Household Longitudinal Survey
PSQI: Pittsburgh Sleep Quality Index
PGLM: Panel generalized linear model
MLE: Maximum likelihood estimation
