# Supplementary material for: Sleep problems among sexual minorities: a longitudinal study on the influence of the family of origin and chosen family
Source: BMC Public Health. 2021 Dec 21;21:2267. doi: 10.1186/s12889-021-12308-0 (PMC8690990; doi:10.1186/s12889-021-12308-0)
Supplement: Supplementary file 1 — Additional file 1. [file 12889_2021_12308_MOESM1_ESM.docx]

Supplementary Table 1: Relative risk and 95% C.I for unadjusted associations between sleep outcomes and family support

|  | Model 1a: Sleep Dysfunction (≧1 sleep problem) | Model 2a: Sleep Duration (<6 hours of sleep) | Model 3a: Sleep Quality (poor sleep quality) |
| --- | --- | --- | --- |
| **Family support (z-score)** | 0.93 (0.89 - 0.96)*** | 0.89 (0.84 - 0.94) *** | 0.92 (0.87 - 0.96) *** |

**p<0.05, **p<0.01, ***p<0.001*

Supplementary Table 2: Relative risk and 95% C.I for unadjusted associations between sleep outcomes and friend support

|  | Model 1a: Sleep Dysfunction (≧1 sleep problem) | Model 2a: Sleep Duration (<6 hours of sleep) | Model 3a: Sleep Quality (poor sleep quality) |
| --- | --- | --- | --- |
| **Friend support (z-score)** | 0.93 (0.89 - 0.97)** | 0.90 (0.85 - 0.96) ** | 0.94 (0.88 - 0.98) * |

**p<0.05, **p<0.01, ***p<0.001*
